# Associations Between Nutritional Status, Cognitive Performance, and Surrogate Metabolic Profiles in School-Aged Children

**DOI:** 10.3390/nu18132040

**Published:** 2026-06-23

**Authors:** Jessica Jazmín Gordillo-Castañeda, Karen Sinaí Xicotencatl-Quintero, Eunice D. Farfán-García, Betsabé Jiménez Ceballos, Dulce María Meneses-Ruiz, Erick Martínez-Herrera, Paola Berenice Zárate-Segura, Arely Vergara-Castañeda, Claudia Erika Fuentes-Venado, Rodolfo Pinto-Almazán

**Affiliations:** 1 Sección de Estudios de Posgrado e Investigación, Escuela Superior de Medicina, Instituto Politécnico Nacional, Plan de San Luis y Díaz Mirón s/n, Mexico City 11340, Mexico; nutriajaz@gmail.com (J.J.G.-C.); kerrquintero@gmail.com (K.S.X.-Q.); erickmartinez_69@hotmail.com (E.M.-H.); 2 Academia de Bioquímica, Sección de Estudios de Posgrado e Investigación, Escuela Superior de Medicina, Instituto Politécnico Nacional, Plan de San Luis y Díaz Mirón s/n, Mexico City 11340, Mexico; farfan800820@gmail.com; 3 Clínica de Trastornos del Sueño, Universidad Autónoma Metropolitana, Unidad Iztapalapa (UAM-I), Av. San Rafael Atlixco 185, Col. Leyes de Reforma, Iztapalapa, Mexico City 09340, Mexico; betsabe.jmz@gmail.com; 4 Noncommunicable Diseases Research Group, Universidad La Salle-México, Benjamín Franklin 45, Mexico City 06140, Mexico; dulce.meneses@lasalle.mx; 5 Fundación Vithas, Grupo Hospitalario Vithas, 28043 Madrid, Spain; 6 Laboratorio de Medicina Traslacional, Sección de Estudios de Posgrado e Investigación, Escuela Superior de Medicina, Instituto Politécnico Nacional, Plan de San Luis y Díaz Mirón s/n, Mexico City 11340, Mexico; pbzars@yahoo.com; 7 Promotion and Education for Health and Food Research Group, Universidad La Salle Mexico, Benjamin Franklin 45, Colonia Condesa, Mexico City 06140, Mexico; arely.vergara@lasalle.mx; 8 Servicio de Medicina Física y Rehabilitación, Hospital General de Zona No 197, Texcoco 56108, Mexico

**Keywords:** malnutrition, pediatric obesity, Wechsler scales, nutritional status, biomarkers, surrogate metabolic indices

## Abstract

**Background**: Childhood malnutrition, manifesting as both underweight and obesity, is a global health concern with potential repercussions on neurodevelopment and metabolic health. **Objective**: To analyze the relationship between nutritional status, metabolic biomarkers, and cognitive performance in school-aged children. **Methods**: A cross-sectional study was conducted with 100 children between 6 and 12 years of age from a public elementary school in the municipality of Chiconcuac de Juárez, Mexico. Participants were categorized according to BMI: underweight (UW), normal weight (NW), overweight (OW), and obesity (OB). Anthropometric evaluation, serum biochemical markers, and three surrogate metabolic indices, namely the Triglyceride–Glucose (TyG), Triglyceride/high-density lipoprotein cholesterol (TG/HDL), and TyG-Body Mass Index (TyG-BMI), were calculated. Cognitive performance was assessed using the Wechsler Intelligence Scale for Children (WISC-IV). **Results**: The OB group children showed significantly higher levels of TG, TC and LDL-C, as well as elevated levels of TyG, TG/HDL and TyG-BMI indices (*p* < 0.05) and lower HDL-C concentration. While no significant differences were found in Full-Scale IQ (FSIQ), the NW group showed significantly higher performance in the PSI compared to all other groups outside the healthy weight range after FDR correction. Spearman’s correlation showed that surrogate metabolic indices exhibited exclusive negative correlations with the PSI in unadjusted bivariate models. **Conclusions**: The extremes of the nutritional status spectrum (UW and OB) are concurrently associated with early metabolic alterations and latent cardiovascular risk, while concurrently tracking with lower performance in selective fluid cognitive domains within unadjusted models. Furthermore, surrogate metabolic indices were shown to be valuable tools that co-vary with neurocognitive profiles.

## 1. Introduction

Malnutrition is defined as a deficiency or imbalance of nutrients, resulting from either a lack of proteins, minerals and fiber (undernutrition, UW), or an excess of fats and carbohydrates (overweight, OW; and obesity, OB). It is a multifactorial phenomenon involving physiological variables, sociocultural patterns, and socioeconomic factors [1,2,3,4]. According to the World Health Organization (WHO), it is estimated that in children under five years of age approximately 150 million suffer from stunted growth, 45 million from wasting, as well as 35.5 million from OW and OB [2,3].

Various biochemical markers reflect the impact of malnutrition, which destabilizes metabolic and immunological axes, as well as the hormonal profile during childhood [5]. In addition to the conventional biochemical profile, metabolic indices have been employed; by relating two or more biochemical or anthropometric parameters, these indices allow for a systemic evaluation of the physiological alterations caused by childhood malnutrition. Recent studies highlight the diagnostic utility of the Triglyceride–Glucose (TyG) Index, the Triglyceride/high-density lipoprotein cholesterol (TG/HDL) ratio, and the TyG-Body Mass Index (BMI) as clinical markers used to evaluate cardiovascular risk and insulin resistance (IR) and the risk of developing chronic pathologies at early ages [6,7,8].

During the transition from early childhood to middle childhood, the nervous system undergoes critical periods of maturation involving morphophysiological changes such as cell proliferation, changes in neuronal plasticity, cerebral myelination, neurotransmitter and hormone metabolism; these have broad implications for behavioral and cognitive development. Since the metabolic profile is disrupted in childhood malnutrition, the availability of energy and structural substrates for the central nervous system is compromised, which can lead to neuroinflammation and alterations in neural circuits. Consequently, malnutrition can have lasting effects on academic performance, behavior, and future productivity [4,9,10,11,12,13,14,15].

Therefore, the objective of this study was to analyze the possible relationship between nutritional states, metabolic biomarkers and cognitive processes in children from 6 to 12 years of age. This was examined through anthropometric assessments and standardized intellectual ability tests. Our hypothesis is that malnutrition, either due to deficit or excess, will cause an altered metabolic profile in children (specifically in the panel of surrogate metabolic indices: TyG, TG/HDL, and TyG-BMI), which will correlate with modifications in specific domains such as working memory, attention, reasoning and processing speed as well as global cognitive performance.

## 2. Materials and Methods

### 2.1. Participant Selection

A cross-sectional study was conducted with 100 children (both sexes) aged between 6 and 12 years from a public elementary school in the semi-urban municipality of Chiconcuac de Juárez, State of Mexico. Recruitment was carried out through an open invitation to all enrolled students, and a non-probabilistic consecutive sampling method was employed to prevent selection bias. Written informed consent was obtained from parents or guardians, and the children also signed a letter of agreement. Medical history and anthropometric measurements were taken to select participants based on strict clinical criteria. Exclusion criteria included children with a prior diagnosis of psychiatric or neurodevelopmental disorders, chronic systemic diseases, secondary obesity (endocrine or genetic pathologies) or pharmacological treatments (such as corticosteroids or stimulants) that could interfere with metabolic markers or cognitive performance. Also, participants with incomplete anthropometric, biochemical, or cognitive evaluations were excluded from the final analysis. The study was conducted in accordance with the Declaration of Helsinki, and the protocol was approved by the Universidad La Salle-México Research and Research Ethics Committees (CIE-2016-6) and by the HRAEI Research and Research Ethics Committees (NR-027-2017).

### 2.2. Anthropometric Assessment and Nutritional Status Classification

Nutritional status was assessed through anthropometry, measuring weight and height with a mechanical column scale with a stadiometer (Seca 700^®^, Seca GmbH & Co. KG, Hamburg, Germany). WHO AnthroPlus software (version 1.0.4) was used to calculate Z-scores for height-for-age (H/A) and Body Mass Index-for-age (BMI/A), according to sex and age. Participants were classified into four categories following WHO criteria: underweight (UW, Z < −2 SD), normal weight (NW, −2 ≤ Z ≤ +1 SD), overweight (OW, +1 < Z ≤ +2 SD), and obesity (OB, Z > +2 SD) [16,17,18].

### 2.3. Blood Sample

On the following day, blood samples (5 mL) were collected from each participant via venipuncture into gold-top serum separator tubes (BD Vacutainer^®^ SST™, Franklin Lakes, NJ, USA). All samples were obtained by trained personnel following an overnight fast of at least 8 h. After collecting, the tubes were allowed to clot at room temperature for 30 min and then centrifuged at 3000 rpm for 10 min at room temperature to obtain the serum. The resulting serum was labeled with the child’s ID and stored at −80 °C until biochemical analysis.

### 2.4. Metabolic Biomarkers Determination

Serum glucose (Glu), Total Cholesterol (TC), triacylglycerides (TG), low-density lipoprotein cholesterol (LDL-C) and high-density lipoprotein cholesterol (HDL-C) were determined in duplicate via spectrophotometry and the mean value was taken for further analysis. Quantification was performed according to the instructions provided by Teco Diagnostics (Anaheim, CA, USA).

#### Biochemical Indexes Calculation

To assess metabolic status and IR, the indexes were calculated as follows:

- Triglyceride–Glucose (TyG) Index: ln [TG (mg/dL) × Glu (mg/dL)/2] [19].
- BMI: Weight (kg)/height (m^2^) [20].
- TyG-BMI: TyG Index × Body Mass Index [20].
- TG/HDL Ratio: Fasting TG (mg/dL)/HDL-C (mg/dL) [21].

### 2.5. Cognitive Assessment

After offering them breakfast, each participant’s cognitive performance was evaluated individually using the Wechsler Intelligence Scale for Children, Fourth Edition (WISC-IV), standardized for the Mexican population. The scale was administered by trained personnel in a quiet, controlled environment to minimize distractions. To intrinsically control for potential cognitive biases driven by age differences, all raw scores were converted into standardized percentiles adjusted strictly for chronological age ranges using validated normative tables. Assessment sessions lasted approximately 60 to 90 min.

WISC-IV is a tool that evaluates intellectual performance as a multidimensional construct, assessing four different cognitive domains through composite indexes and Full-Scale total score:

1. Verbal Comprehension Index (VCI): Evaluates verbal reasoning and comprehension, and the ability to process verbal stimuli, suggesting accumulated knowledge and language skills.
2. Perceptual Reasoning Index (PRI): Examines non-verbal fluid reasoning, logical relationships, and spatial processing to solve visual problems.
3. Working Memory Index (WMI): Determines attention span, concentration and short-term memory required to temporarily retain information.
4. Processing Speed Index (PSI): Determines mental efficiency and automatic competences concerning visual attention and rapid execution.
5. Full-Scale Intelligence Quotient (FSIQ): Calculates overall cognitive functioning.

Raw scores were converted into age-corrected standardized assessments (percentiles and composite scores) according to the manual [22].

### 2.6. Statistical Analysis

Data analysis was performed using Prism 9.3.0 software (GraphPad, San Diego, CA, USA). Data distribution was assessed using the Shapiro–Wilk test. One-way ANOVA followed by Tukey’s post hoc test was performed to identify group differences for normally distributed data. Kruskal–Wallis test followed by Dunn’s post hoc test for non-normally distributed data was used. Normally distributed serum biomarkers were presented as mean ± standard deviation (SD), while non-normally distributed data were expressed as median and interquartile range (IQR, P25–P75 percentiles).

Results for the WISC-IV scale indexes (VCI, PRI, WMI, PSI, and FSIQ) were expressed as percentile rank and results represented through box-and-whisker plots, superimposing every individual patient’s score as a jittered data point. Considering the non-normal distribution of variables, Spearman’s rank correlation coefficients (*rs*) were used to evaluate the association between serum biochemical biomarkers and cognitive subtests from the WISC-IV values. As multiple simultaneous comparisons across cognitive domains were performed, Benjamini–Krieger–Yekutieli false discovery rate (FDR) at 10% (Q = 0.10) was applied. Conventional *p* < 0.05 values were considered statistically significant and FDR-corrected q-values throughout and establish conclusions on results that remain significant after correction. All analyses were stratified by weight status (UW, NW, OW, and OB).

## 3. Results

### 3.1. Sociodemographic Analysis

Table 1 presents the sociodemographic profile of the study participants. No statistically significant differences were observed between the nutritional groups regarding age (*p* = 0.5232) or sex (*p* = 0.9907). Neither the education of the primary caregiver (*p* = 0.6062) nor the marital status (*p* = 0.3035) presented significant variations between the groups.

### 3.2. Metabolic Biomarkers Analysis

**Figure 1:**
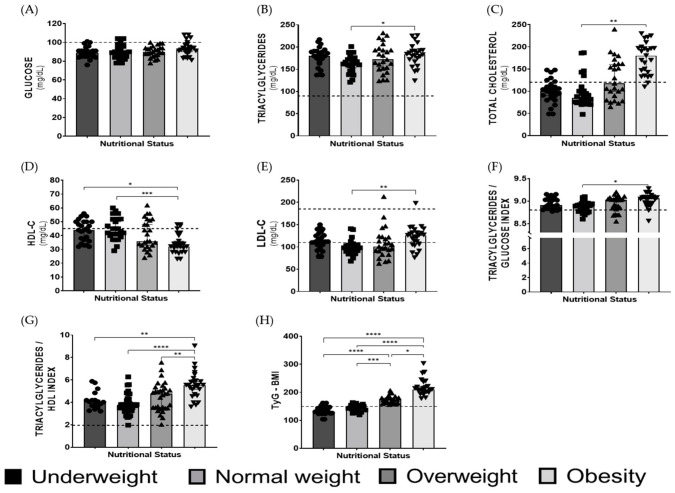
Comparison of serum biochemical markers and surrogate metabolic indices by nutritional status (*n* = 100). Data are presented as mean ± standard deviation (SD) for normally distributed variables, or as median (interquartile range, P25–P75) for non-normally distributed variables. (**A**) Glucose (GLU); (**B**) Triacylglycerides (TG); (**C**) Total Cholesterol (TC); (**D**) high-density lipoprotein cholesterol (HDL-C); (**E**) low-density lipoprotein cholesterol (LDL-C); (**F**) Triacylglyceryde/Glucose Index (TyG); (**G**) Triglyceride/HDL-C Ratio (TG/HDL-C Index); (**H**) TyG-Body Mass Index (TyG-BMI). Statistical significance differences were represented as follows: * *p* < 0.05, ** *p* < 0.01, *** *p* < 0.001, and **** *p* < 0.0001. Normal values for children: Fasting Glucose < 100 mg/dL, TG < 90 mg/dL, TC < 170 mg/dL, LDL < 110 mg/dL, HDL > 45 mg/dL, TG/HDL-C ratio < 2.0, TyG < 8.85 and TyG-BMI < 140.

Figure 1 presents the biochemical data of the UW, NW, OW, and OB children analyzed in this study. Statistically significant differences were found between groups in the concentrations of TG, TC, HDL, LDL, and the TyG Index. Participants in the OB group showed significantly higher levels of TG (>90 mg/dL), TC (>170 mg/dL), and LDL (>110 mg/dL) compared to the NW group. Likewise, a significant reduction in HDL levels (<45 mg/dL) was observed in the OB group relative to the NW and UW groups. Regarding the calculated indices, the OB group showed significantly higher values for TyG, TG/HDL, and TyG-BMI compared to the NW group. Furthermore, TG/HDL and TyG-BMI indices were significantly higher in the OB group compared to children in the UW and OW groups.

### 3.3. Cognitive Assessment Analysis

**Figure 2:**
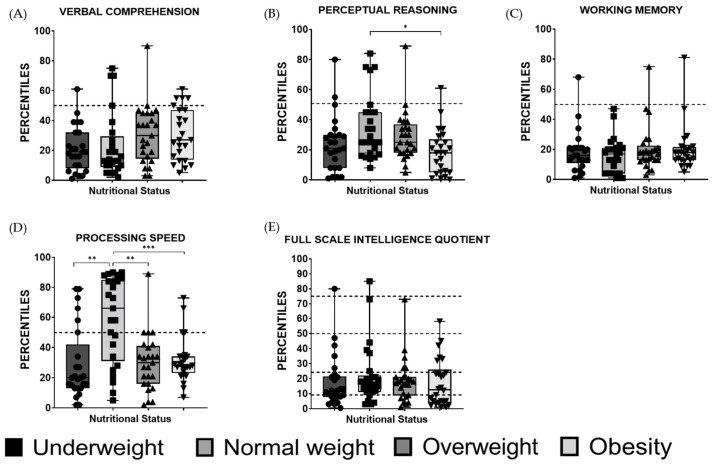
Data are presented as boxplots showing medians, interquartile ranges (IQR), and minimum/maximum values. Statistical significance differences were represented as follows: * *p* < 0.05, ** *p* < 0.01, and *** *p* < 0.001. Psychometric classifications: average percentile = P25–P74, low average = P9–P24, borderline = P2–P8.

Figure 2 shows the percentile ranks obtained from the WISC-IV test for the UW, NW, OW, and OB groups. A significant difference was found in the Perceptual Reasoning Index (PRI) for the OB group compared to the NW group, with scores falling below the 50th percentile (P50). Additionally, significant differences were observed in the Processing Speed Index (PSI), where the NW group exhibited higher scores relative to the UW, OW, and OB groups. This core difference remained robust after strict FDR correction. Conversely, subgroup-specific correlations that did not survive multiple-testing adjustment are presented strictly as nominal findings in Table 2.

### 3.4. Spearman Correlation Analysis

Spearman correlation analysis (Table 2) was performed to determine the associations between metabolic parameters and indexes (GLU, TC, HDL, LDL, TG, TyG, TG/HDL, and TyG-BMI) and the WISC-IV indexes (VCI, WMI, PRI, PSI, and FSIQ).

In the NW group (Table 2), the VCI showed significant negative correlations with LDL (*rs* = −0.5052, *p* = 0.0478) and the TG/HDL ratio (*rs* = −0.5441, *p* = 0.0316), while a positive correlation was found with the TyG-BMI (*rs* = 0.6393, *p* = 0.0122). Regarding the PRI, it correlated positively with HDL (*rs* = 0.7567, *p* = 0.0027) and negatively with the TG/HDL ratio (*rs* = −0.5560, *p* = 0.0346). The WMI exhibited negative associations with LDL (*rs* = −0.7333, *p* = 0.0202) and TG/HDL (*rs* = −0.6970, *p* = 0.0306). Furthermore, PSI was significantly associated with TC (*rs* = 0.5882, *p* = 0.0147), TG/HDL (*rs* = −0.4975, *p* = 0.0442), and the TyG Index (*rs* = −0.6348, *p* = 0.0074). Finally, FSIQ showed significant correlations with LDL (*rs* = −0.5442, *p* = 0.0381) and TG/HDL (*rs* = −0.5924, *p* = 0.0222).

In the UW group (Table 2), the PRI was negatively correlated with TC (*rs* = −0.6575, *p* = 0.0172), TG/HDL (*rs* = −0.7417, *p* = 0.0051), and TyG-BMI (*rs* = −0.5934, *p* = 0.0360), while showing a strong positive correlation with HDL (*rs* = 0.7752, *p* = 0.0027). The PSI exhibited negative correlations with GLU (*rs* = −0.6224, *p* = 0.0347), TC (*rs* = −0.7333, *p* = 0.0202), and TG/HDL (*rs* = −0.7692, *p* = 0.0049). Additionally, FSIQ was negatively associated with TC (*rs* = −0.6758, *p* = 0.0137) and positively with HDL (*rs* = 0.8308, *p* = 0.0007).

For the OW group (Table 2), a significant positive correlation was found between PRI and the TG/HDL ratio (*rs* = 0.4800, *p* = 0.0322). Additionally, WMI was negatively correlated with TC (*rs* = −0.6834, *p* = 0.0240). PSI showed significant negative associations with both TG (*rs* = −0.5287, *p* = 0.0372) and TC (*rs* = −0.6608, *p* = 0.0120). Finally, FSIQ showed significant negative correlations with HDL (*rs* = −0.6859, *p* = 0.0031) and the TG/HDL ratio (*rs* = −0.4876, *p* = 0.0401).

In the OB group (Table 2), the VCI showed a significant negative correlation with the TyG-BMI (*rs* = −0.5359, *p* = 0.0284), while FSIQ was negatively correlated with LDL (*rs* = −0.5180, *p* = 0.0418). Conversely, significant positive correlations were observed between HDL and PSI (*rs* = 0.6743, *p* = 0.0265), as well as between HDL and WMI (*rs* = 0.6433, *p* = 0.0201) and FSIQ (*rs* = 0.5269, *p* = 0.0460).

#### Spearman Correlation Analysis of Surrogate Metabolic Indexes and Cognitive Performance Beyond Nutritional Status

Finally, a Spearman correlation analysis (Table 3) was performed on the entire study population (*n* = 100) to evaluate the associations between surrogate metabolic indexes and WISC-IV indexes regardless of nutritional status groups. Remarkably, all three surrogate metabolic indexes showed selective, negative, and significant correlation with PSI. No other statistically significant correlations were observed between the metabolic indexes and the remaining VCI, PRI, WMI, and FSIQ.

## 4. Discussion

Malnutrition is a multifactorial issue that co-occurs with complex alterations in physical, metabolic, and cognitive development in children. Globally, millions of children are affected by UW, OW, and OB—conditions frequently tied to biochemical alterations that correlate with variations in brain structure and function, including processes like memory, attention, reasoning, and academic performance. As in previous studies, OB children presented significant metabolic changes. Specifically, we observed high levels of TC, TG, and LDL-C cholesterol as well as elevated TyG, TG/HDL, and TyG-BMI indexes, in conjunction with a decrease in HDL-C [5,6,7,8].

Regarding cognitive performance, although no significant differences were observed among groups in the standardized percentiles adjusted strictly by age for VCI, WMI, or FSIQ, the NW group showed higher performance on the PSI compared to the other nutritional groups. This result highlights that changes in the groups outside the healthy weight range are associated with lower processing speed. Notably, all groups scored below the 50th percentile (P50) in almost all the cognitive domains. Furthermore, correlation analyses, already stratified by nutritional status, as well as the overall correlation, expanded upon these findings by emphasizing statistical co-variations between these cognitive metrics and early metabolic changes. While these inverse associations clustered most prominently within the OB group, it is important to mention that mainly the correlations related to PSI remained significant after FDR correction, whereas other subgroup trends must be interpreted strictly as tentative, exploratory trends within models unadjusted for sex, socioeconomic status, or parental education. In concordance, the assessment of the entire population revealed a remarkably consistent and robust pattern: indirect metabolic indices exhibited an exclusive and significant inverse trend with lower processing speed performance. The present data suggest that child malnutrition, specifically OB groups, co-occurs with metabolic changes and low scores in specific domains of cognition, positioning processing speed as a highly sensitive and selective cognitive domain associated with early subclinical metabolic risk in the pediatric population. In the WISC-IV, individual subscales like the PSI represent a single component scale of the FSIQ. Therefore, metabolic alterations may show a concurrent association first in these individual domains within unadjusted bivariate models. Speculatively, prolonged metabolic fluctuations over time might progressively involve additional functional paths, eventually compromising the total FSIQ performance later in development.

Even into middle childhood, the nervous system undergoes critical periods of maturation. For example, synaptic density reaches adult levels, important phases of myelination are completed in the frontal lobes, and gray matter reaches its maximum volume and refinement. These neurobiological processes support the development of complex functions such as language, reading, and memory [23,24]. Accordingly, inadequate nutritional intake, whether due to excess or deficiency of substrates, during this stage shows a consistent statistical concurrence with changes in brain maturation and lower cognitive performance. [4,10,11,12,13,14,15,25,26,27]. In this regard, Aguayo et al. and Pollitt et al. concluded that children who skip breakfast have greater difficulties with concentration and information processing, regardless of their weight status [28,29]. Similarly, Li et al. highlighted in their study that UW school-aged children present lower cognitive abilities, which concurrently traces with lower PSI [30], while Waruiru et al. correlated UW in early stages to poorer long-term neurocognitive development [31].

Regarding GLU levels, although no significant differences were observed among groups, a nominal negative correlation was identified in the UW group concerning PSI. These findings align with Shapiro et al., who observed that increased serum glucose co-occurs with a reduction in cognitive flexibility [32]. Nonetheless, because this result did not survive FDR correction, it must be treated strictly as a tentative, unadjusted trend subject to potential residual confounding from sociodemographic factors.

Furthermore, scientific evidence reveals that diets with an excessive intake of lipids and carbohydrates frequently parallel systemic inflammatory processes and co-vary with changes in brain regions associated with executive functions [33]. González-Palacios et al. also concluded that OW is negatively associated with cognitive function, highlighting a potential vulnerability in intellectual performance that requires cautious interpretations [34].

Regarding lipid biomarkers, contradictory evidence exists in the literature. While severe malnutrition often results in reduced serum lipids, children with mild malnutrition or UW may show increased TC and LDL-C, along with decreased HDL-C. Notably, TG levels are consistently elevated in cases of UW, acute, and chronic malnutrition [35,36,37,38,39,40,41]. On the other hand, Umer et al. reported that childhood OB is positively associated with increased TG and inversely with HDL-C, suggesting an early cardiovascular risk factor. Similarly, pro-inflammatory factor levels (IL-6 and TNF-α) increase in patients with hypercholesterolemia, which is associated with cognitive deficiency [33,42,43,44].

In the present study, all participants had TG levels above the upper limit of acceptable values (>90 mg/dL), with the OB group presenting significantly higher concentrations. A significant negative correlation was also observed in the OW group with respect to the PSI, suggesting that elevated TG levels are inversely associated with processing speed [45]. Even though this co-variation is supported by the global cohort trend analysis, it did not survive FDR correction and must be interpreted strictly as a tentative trend subject to residual confounding. This is consistent with Jung et al. and Hanh et al., who reported that childhood obesity increases the prevalence of hypertriglyceridemia [44,45].

Similarly, OB group TC levels surpassed the AHA recommendations (<170 mg/dL) [46]. While nominal negative correlations were observed between TC and both PRI and WMI in the non-normal weight groups, these associations did not survive FDR control. Notably, the PSI was the most sensitive to statistical co-variations, where lower PSI scores and deviations from healthy weight status (NW group Me = 62.00) concurrently clustered together (UW, OW, and OB groups Me = 27.00), without tracking with changes in overall FSIQ. Moreover, the PSI presented six distinct associations that survived a relaxed FDR threshold (q = 0.0701). In the NW group, the PSI showed a moderate positive correlation with TC (r = 0.5882, *p* = 0.0147). Inversely, the PSI presented a severe negative correlation in the UW and OW groups (r = −0.7622, *p* = 0.0055; and r = −0.6608, *p* = 0.0120; respectively). It is important to mention that all three (NW, UW and OW) PSI correlations survived the q = 0.0701 threshold. These results reflect the importance of alterations in nutritional status, suggesting that the transport of lipids across the blood–brain barrier might be modified, emphasizing a potential factor that might co-vary with fluctuations in processing speed efficiency within unadjusted sociodemographic contexts [46,47,48].

Dyslipidemia combined with malnutrition (UW, OW, and OB) disturbs homeostasis, highlighting its potential statistical concurrence with cognitive functions in the short and long term [47,48]. Current data suggest that elevated TC levels co-vary with lower scores in cognitive processes in school-aged children, consistent with the findings of Suryawan et al. on the associations between weight status and neurocognitive outcomes [49]. Furthermore, Ansuya et al., in their study of malnourished preschool children, determined that the population that received a home-centered feeding intervention had better cognitive development than those that did not receive it [12].

Concerning HDL-C, most participants had values below the recommended threshold (>45 mg/dL), with the NW group being the only one whose mean concentration was adequate [50]. In addition, HDL-C shows strong positive concurrent associations with FSIQ in children outside the healthy weight range after strict FDR correction. Nonetheless, because these models are unadjusted for sociodemographic factors, this finding must be interpreted cautiously due to potential residual confounding. The present data suggest that in pediatric populations suffering malnutrition at both ends, optimal HDL-C concentrations may act as a biological buffer, reducing the systemic metabolic insults that co-occur with variations in specific neurocognitive domains. As for LDL-C, it presents a dual associative pattern. While LDL-C correlates positively with FSIQ in the NW group, it turns into a significant negative correlation in the OB group. This suggests that elevated serum LDL-C concentrations are associated with lower overall cognitive performance only when coupled with high adiposity within unadjusted bivariate strata.

Finally, regarding metabolic indices, several authors highlight their usefulness in identifying a higher risk of IR and chronic metabolic disorders in children [50,51]. In this study, the TyG Index showed significant differences between weight groups, with the OB group displaying a higher risk of IR. Remarkably, all the participants of the study surpassed the normal range (8.6–8.8) [52,53]. Also, a significant negative correlation was identified between the TyG Index and PSI in the NW group (r = −0.6348, *p* = 0.0074) surviving multiple-testing correction (q = 0.0701). This suggests that even slight TyG elevations are inversely associated with PSI scores under healthy weight conditions. In concordance, Rubens et al. and Mangones et al. previously associated changes in executive functions, PSI and visual-spatial processing with IR [54,55].

Likewise, the TG/HDL ratio was among the thresholds in all groups, and statistically higher in the OB group. Remarkably, a significant negative correlation was observed with FSIQ in the UW (r = −0.7143, *p* = 0.0079, q = 0.0631) group. This result is consistent with the AHA report, which associates metabolic markers with cognitive performance in unadjusted models [46].

Moreover, the TyG-BMI exceeded reference values specifically in the OB group, where it was negatively correlated with VCI. In the NW group, the TyG-BMI showed a positive association, suggesting that under homeostatic conditions, physiological metabolic adaptations coexist with optimal verbal abstraction. However, when this index surpasses the optimal threshold in obesity, it reveals a complete inversion from the positive association observed in the NW group. This relationship remained robust after FDR correction, reinforcing the notion that obesity is a key factor associated with altered metabolic dynamics and lower neurocognitive performance [47]. Consistently, González-Palacios et al. concluded that weight gain is associated with a higher vulnerability to executive dysfunction [34]. It is important to note that when the entire study population was analyzed globally, a highly consistent pattern emerged, with all the three indirect metabolic indices being negatively correlated with PSI, with no other relationships observed with the other domains or the FSIQ within unadjusted bivariate models.

Collectively, these findings indicate that malnutrition, especially OB, is associated with metabolic biomarkers and lower performance in critical cognitive domains such as PSI and VCI. Broadly, FDR analysis across all WISC-IV subscales indicates that pediatric cognition does not correlate linearly with metabolism. Instead, specific levels of nutritional biochemical markers correlate with fluctuations in cognitive scores; while the UW group exhibits associations of both PRI and PSI with TC and TG imbalances, OB correlates with lower VCI abilities and IR pathways in unadjusted bivariate models.

### Limitations

Despite the contributions presented in this study, it has some limitations. First, its cross-sectional design prevents the establishment of causal relationships or predictive directionality. Second, although each nutritional status subgroup was meticulously categorized, the sample size per group (*n* = 25) represents a statistical limitation which can increase statistical variability and make correlation coefficients more sensitive to outliers. Moreover, due to this group subdivision, multivariable regressions could not be performed, introducing an inherent risk of residual confounding from transversal cofactors (e.g., sex, caregiver education and socioeconomic background). Nonetheless, cognitive biases from age differences were intrinsically controlled by using percentiles adjusted for chronological age ranges. Because these variables could not be formally controlled, individual correlations must be interpreted strictly as unadjusted statistical associations that require caution. Third, direct quantification of fasting insulin was not performed due to budgetary constraints and the epidemiological nature of our school-based study design; However, our use of validated lipid–glucose surrogate markers reflects standard and low-cost clinical options widely available in the Mexican public health system, supporting the practical translatability of our results. Lastly, the lack of inflammatory markers and serum micronutrients (vitamins and minerals) limits a more comprehensive biological analysis. Finally, although BMI was the primary measure, the addition of other anthropometric measurements (waist or neck circumference, etc.) could have improved the assessment of body fat distribution. Future longitudinal studies with larger cohorts are needed to confirm these associations.

## 5. Conclusions

The extremes of the nutritional status spectrum (UW and OB) are associated with concurrent metabolic alterations, IR and latent cardiovascular risk. Although no significant changes in FSIQ were identified, the findings suggest that these nutritional statuses are selectively associated with specific cognitive domains, such as PRI, PSI, and VCI in unadjusted bivariate models. This study highlights surrogate metabolic indices as valuable tools that correlate with specific neurocognitive profiles. These indices were essential in identifying metabolic alterations that co-fluctuate with lower cognitive efficiency, even in school-age children of NW. These results underscore the relevance of integrating practical clinical metabolic metrics to better understand and identify concurrent variations in neurodevelopment.

## Figures and Tables

**Table 1:** Sociodemographic characteristics of the participants by nutritional status.

Variables	UW (*n* = 25)	NW (*n* = 25)	OW (*n* = 25)	OB (*n* = 25)	*p*-Value
Age (Mean years ± SD)	8.92 ± 1.38	8.47 ± 1.30	8.88 ± 1.33	8.41 ± 1.50	0.5232 ^a^
Sex *n* (%)					0.6237 ^b^
Male	11 (44.00%)	13 (52.00%)	11 (44.00%)	13 (52.00%)	
Female	14 (56.00%)	12 (48.00%)	14 (56.00%)	12 (48.00%)	
Primary Caregiver Education *n* (%)					0.6062 ^b^
Basic Education (≤9 years)	6 (24.00%)	8 (32.00%)	10 (38.46%)	10 (40.00%)	
Upper Secondary (12 years)	16 (64.00%)	11 (44.00%)	12 (46.15%)	10 (40.00%)	
Undergraduate (16 years)	3 (12.00%)	5 (20.00%)	4 (15.39%)	4 (20.00%)	
Postgraduate (>16 years)	0 (0.00%)	1 (4.00%)	0 (0.00%)	0 (0.00%)	
Marital Status *n* (%)					0.3035 ^b^
Married/Cohabiting	3 (12.0%)	8 (32.0%)	8 (30.8%)	5 (20.8%)	
Single/Divorced	22 (88.0%)	17 (68.0%)	18 (69.2%)	19 (79.2%)	

M = mean; SD = standard deviation; *n* = frequency. Abbreviations: UW: underweight; NW: normal weight; OW: overweight; OB: obesity. Nutritional status was classified according to WHO BMI-for-age Z scores. ^a^ *p*-value from one way ANOVA. ^b^ *p*-value from Pearson’s Chi-square.

**Table 2:** Spearman’s correlation between serum biomarkers and WISC-IV cognitive domains according to nutritional status.

WISC-IV Scale	Biochemical Marker	NW (*n* = 25)				UW (*n* = 25)				OW (*n* = 25)				OB (*n* = 25)			
		*rs*	*p*	95% CI	FDR (Q = 10%)	*rs*	*p*	95% CI	FDR (Q = 10%)	*rs*	*p*	95% CI	FDR (Q = 10%)	*rs*	*p*	95% CI	FDR (Q = 10%)
VCI	GLU	0.1635	0.5425	−0.3754–0.6198	No	−0.1376	0.6520	−0.6507–0.4619	No	0.2007	0.4516	−0.3418–0.6429	No	−0.2491	0.3322	−0.6605–0.2774	No
	TG	0.3485	0.2021	−0.2153–0.7381	No	−0.0055	0.9929	−0.5674–0.5599	No	0.1987	0.4579	−0.3437–0.6417	No	0.0774	0.7663	−0.4316–0.5489	No
	TC	−0.0559	0.8369	−0.5481–0.4650	No	−0.2527	0.4043	−0.7146–0.3625	No	0.0839	0.7566	−0.4427–0.5674	No	−0.0829	0.7496	−0.5528–0.4270	No
	HDL	0.2618	0.3262	−0.2837–0.6792	No	0.3857	0.1926	−0.2274–0.7798	No	−0.1982	0.4577	−0.6414–0.3441	No	0.1905	0.4602	−0.3332–0.6244	No
	LDL	−0.5052	0.0478 *	−0.8061–0.003404	No	0.1868	0.5413	−0.4211–0.6789	No	0.1177	0.6621	−0.4148–0.5902	No	−0.0751	0.7811	−0.5614–0.4498	No
	TG/HDL	−0.5441	0.0316 *	−0.8061–0.003404	No	−0.3956	0.1822	−0.7843–0.2162	No	0.2561	0.3357	−0.2893–0.6759	No	0.0233	0.9298	−0.4746–0.5099	No
	TyG	0.2324	0.3852	−0.3122–0.6620	No	0.3736	0.2095	−0.2407–0.7742	No	0.1781	0.5066	−0.3624–0.6289	No	−0.0908	0.7272	−0.5583–0.4205	No
	TyG-BMI	0.6393	0.0122 *	0.1727–0.8715	No	0.1209	0.6960	−0.4751–0.6408	No	−0.0471	0.8627	−0.5419–0.4719	No	−0.7505	0.0008 *	−0.9075–−0.4092	0.0273
PRI	GLU	−0.2527	0.3825	−0.6997–0.3364	No	−0.2283	0.4500	−0.7017–0.3848	No	−0.1664	0.5502	−0.6354–0.3924	No	0.0528	0.8394	−0.4514–0.5315	No
	TG	0.2132	0.4636	−0.3730–0.6777	No	0.3274	0.2731	−0.2897–0.7522	No	0.2696	0.2373	−0.1966–0.6363	No	−0.1006	0.6993	−0.5651–0.4123	No
	TC	0.1912	0.5121	−0.3926–0.6652	No	−0.6575	0.0172 *	−0.8909–−0.1492	No	−0.0580	0.8027	−0.4882–0.3948	No	0.1891	0.4639	−0.3345–0.6235	No
	HDL	0.5659	0.0473 *	0.003403–0.8564	0.0842	0.7752	0.0027 *	0.3757–0.9317	No	−0.0783	0.7359	−0.5036–0.3775	No	0.4325	0.1225	−0.1444–0.7900	No
	LDL	0.2662	0.3546	−0.3236–0.7070	No	0.2967	0.3247	−0.3205–0.7371	No	0.4007	0.0719	−0.05115–0.7163	No	−0.1525	0.5691	−0.6128–0.3850	No
	TG/HDL	−0.5560	0.0419 *	−0.8442–−0.01866	0.0842	−0.7418	0.0051 *	−0.9205–−0.3061	No	−0.5170	0.0196 *	−0.7863–−0.08261	No	−0.0037	0.9905	−0.4952–0.4897	No
	TyG	0.2324	0.3852	−0.5922–0.4898	No	0.0440	0.8917	−0.5329–0.5929	No	0.1707	0.4595	−0.2943–0.5703	No	−0.1816	0.4826	−0.6187–0.3414	No
	TyG-BMI	0.6393	0.0122 *	−0.7918–0.1974	No	−0.5934	0.036 *	−0.8670–−0.04476	No	0.0958	0.6797	−0.3623–0.5166	No	−0.0994	0.7027	−0.5642–0.4133	No
WMI	GLU	−0.2000	0.5837	−0.7251–0.5105	No	0.2032	0.5205	−0.4354–0.7058	No	−0.0636	0.8603	−0.6511–0.5715	No	−0.3928	0.1837	−0.7831–0.2194	No
	TG	0.0303	0.946	−0.3480–0.8070	No	0.2347	0.4601	−0.4082–0.7220	No	−0.2727	0.4181	−0.7587–0.4084	No	0.1562	0.5912	−0.4227–0.6446	No
	TC	−0.1273	0.7330	−0.5878–0.6681	No	−0.1185	0.7281	−0.6818–0.5331	No	−0.6834	0.024 *	−0.9136–−0.1214	No	0.2772	0.3348	−0.3129–0.7129	No
	HDL	−0.7000	0.0433 *	−0.7412–0.4841	No	0.2837	0.3690	−0.3635–0.7462	No	−0.2323	0.4889	−0.7398–0.4437	No	0.6433	0.0201 *	0.1249–0.8857	No
	LDL	−0.7333	0.0202 *	−0.9129–−0.06304	No	0.3468	0.2679	−0.3012–0.7757	No	−0.1091	0.7545	−0.6767–0.5398	No	−0.0937	0.7595	−0.6243–0.4961	No
	TG/HDL	−0.6970	0.0306 *	−0.9092–−0.04132	No	0.2067	0.5168	−0.4325–0.7076	No	0.0182	0.9673	−0.6013–0.6241	No	−0.0418	0.8882	−0.5718–0.5129	No
	TyG	−0.6121	0.0667	−0.8873–0.07247	No	0.3433	0.2726	−0.3048–0.7741	No	−0.2273	0.5034	−0.7374–0.4479	No	0.0286	0.9243	−0.5225–0.5629	No
	TyG-BMI	0.4667	0.1786	−0.3082–0.8220	No	0.0385	0.9083	−0.5609–0.6114	No	−0.2273	0.5034	−0.7374–0.4479	No	−0.3168	0.2679	−0.7336–0.2732	No
PSI	GLU	−0.3568	0.1594	−0.7224–0.1645	No	−0.6224	0.0347 *	−0.8857–−0.05618	No	0.0446	0.8606	−0.4434–0.5121	No	−0.0803	0.7942	−0.6161–0.5062	No
	TG	0.1324	0.6120	−0.3852–0.5866	No	−0.1399	0.6673	−0.8857–−0.05618	No	−0.5287	0.0372 *	−0.8171–−0.02869	No	−0.1684	0.5989	−0.6872–0.4642	No
	TC	0.5882	0.0147 *	0.1348–0.8380	0.0701	−0.7622	0.0055 *	−0.9321–−0.3175	0.0701	−0.6608	0.012 *	−0.8859–−0.1837	0.0701	−0.2102	0.5088	−0.7094–0.4295	No
	HDL	−0.6118	0.0136 *	−0.4699–0.5144	0.0701	0.3427	0.2762	−0.3055–0.7738	No	−0.0969	0.7021	−0.5499–0.4001	No	0.6743	0.0265 *	0.1047–0.9108	No
	LDL	0.4534	0.0693	−0.05025–0.7732	No	−0.2657	0.4042	−0.7375–0.3803	No	−0.2909	0.2416	−0.6754–0.2179	No	−0.3053	0.3322	−0.7565–0.3429	No
	TG/HDL	−0.4975	0.0442 *	−0.7952–−0.006735	No	−0.7692	0.0049 *	−0.9343–−0.3327	0.0701	−0.2205	0.3793	−0.6323–0.2884	No	0.1541	0.6129	−0.4485–0.6603	No
	TyG	−0.6348	0.0074 *	−0.8588–−0.2071	0.0701	−0.4266	0.1689	−0.8104–0.2136	No	−0.2816	0.2577	−0.6698–0.2276	No	−0.0385	0.9022	−0.5893–0.5368	No
	TyG-BMI	−0.2966	0.2470	−0.6885–0.2294	No	−0.1818	0.5731	−0.6944–0.4532	No	−0.2246	0.3702	−0.6349–0.2844	No	−0.2692	0.3734	−0.7231–0.3470	No
FSIQ	GLU	−0.4021	0.1377	−0.7652–0.1551	No	0.0193	0.9533	−0.5503–0.5766	No	−0.0290	0.9033	−0.4384–0.5167	No	−0.0331	0.8964	−0.5036–0.4526	No
	TG	0.4111	0.1284	−0.1446–0.7696	No	−0.011	0.9744	−0.5711–0.5561	No	−0.2090	0.3906	−0.6171–0.3437	No	−0.0413	0.8707	−0.5097–0.4460	No
	TC	0.0714	0.8025	−0.4707–0.5744	No	−0.6758	0.0137 *	−0.8976–−0.1812	0.0814	−0.0299	0.9034	−0.6295–0.3258	No	−0.0362	0.8867	−0.5059–0.4501	No
	HDL	−0.0984	0.7259	−0.5923–0.4493	No	0.8308	0.0007 *	0.5025–0.9497	0.0208	0.6859	0.0031 *	0.2921–0.8808	0.0460	0.5269	0.0458 *	0.0033–0.8237	No
	LDL	0.5442	0.0381 *	0.02753–0.8314	No	0.1074	0.7259	−0.4856–0.6327	No	0.0783	0.7430	−0.3184–0.6120	No	−0.5180	0.0418 *	−0.8121–−0.01397	No
	TG/HDL	−0.5929	0.0222 *	−0.4594–0.5840	No	−0.7143	0.0079 *	−0.9111–−0.2522	0.0631	0.4876	0.0401 *	−0.7773–0.0035	No	−0.0764	0.7632	−0.5353–0.4173	No
	TyG	−0.3786	0.1649	−0.4679–0.5768	No	−0.0879	0.7784	−0.6208–0.5005	No	−0.1497	0.5286	−0.5375–0.4148	No	−0.1260	0.6184	−0.5701–0.3751	0.0631
	TyG-BMI	0.5393	0.0406 *	−0.3193–0.6827	No	−0.0604	0.8491	−0.6035–0.5209	No	−0.1242	0.602	−0.5679–0.3779	No	−0.5996	0.0085	−0.8377–−0.1698	No

Spearman’s rank correlation coefficient. Data represents the association between serum biochemical biomarkers and WISC-IV cognitive performance. Abbreviations: UW: underweight; NW: normal weight; OW: overweight; OB: obesity. Cognitive Indices: VCI: Verbal Comprehension Index; PRI: Perceptual Reasoning Index; WMI: Working Memory Index; PSI: Processing Speed Index; FSIQ: Full-Scale Intelligence Quotient. Biochemical markers: GLU: Glucose; TG: Triglycerides; TC: Total Cholesterol; HDL: High-Density Lipoprotein; LDL: Low-Density Lipoprotein; TG/HDL: Triglyceride-to-HDL ratio; TyG: Triglyceride–Glucose Index; TyG-BMI: Triglyceride–Glucose/Body Mass Index. Weight groups: Categorized by BMI according to WHO criteria (UW, NW, OW, and OB). Asterisks indicate statistical significance for the correlation: * *p* < 0.05.

**Table 3:** Global Spearman’s correlation between surrogate metabolic indices and cognitive domains.

Surrogate Metabolic Index	VCI (*n* = 100)			PRI (*n* = 100)			WMI (*n* = 100)			PSI (*n* = 100)			FSIQ (*n* = 100)		
	*rs*	*p*	95% CI	*rs*	*p*	95% CI	*rs*	*p*	95% CI	*rs*	*p*	95% CI	*rs*	*p*	95% CI
TG/HDL	0.0895	0.3617	−0.1087–0.2808	−0.0217	0.8251	−0.2170–0.1753	0.0972	0.3218	−0.1010–0.2879	−0.3201	0.0013 *	−0.4921–−0.1241	−0.0103	0.9166	−0.2061–0.1863
TyG	0.0507	0.6165	−0.1529–0.2502	0.1491	0.1309	−0.05055–0.3372	0.0452	0.6473	−0.1534–0.2402	−0.3576	0.0003 *	−0.5243–−0.1645	−0.0158	0.8725	−0.2114–0.1810
TyG-BMI	−0.0172	0.8621	−0.2136–0.1806	−0.0969	0.3231	−0.2877–0.1013	0.0892	0.3632	−0.1090–0.2805	−0.3438	0.0004 *	−0.5089–−0.1544	0.0108	0.9122	−0.1858–0.2066

VCI: Verbal Comprehension Index; PRI: Perceptual Reasoning Index; WMI: Working Memory Index; PSI: Processing Speed Index; FSIQ: Full-Scale Intelligence Quotient. Statistically significant correlations, Asterisks indicate statistical significance for the correlation: * *p* < 0.05.

## Data Availability

The data presented in this study are available on request from the corresponding author due to ethical and privacy restrictions.

## Abbreviations

UW	Underweight
NW	Normal weight
OW	Overweigh
OB	Obesity
Glu	Glucose
TC	Total cholesterol
TG	Triacylglycerides
LDL-C	low-density lipoprotein cholesterol
HDL-C	high-density lipoprotein cholesterol
TyG	Triglyceride–Glucose Index
TG/HDL ratio	Triglyceride / HDL ratio
TyG-BMI	Triglyceride–Glucose Index x Body Mass Index
WISC-IV	Wechsler Intelligence Scale for Children, Fourth Edition
VCI	Verbal Comprehension Index
PRI	Perceptual Reasoning Index
WMI	Working Memory Index
PSI	Processing Speed Index
FSIQ	Full-Scale Intelligence Quotient
SD	standard deviation
IQR, P25–P75	Interquartile range Percentile 25–Percentile 75
